# Reduced Graphene Oxide-Coated Iridium Oxide as a Catalyst for the Oxygen Evolution Reaction in Alkaline Water Electrolysis

**DOI:** 10.3390/molecules30092069

**Published:** 2025-05-07

**Authors:** Shengyin Luo, Ziqing Zuo, Hongbin Sun

**Affiliations:** 1 College of Sciences, Northeastern University, Shenyang 110819, China; 2210055@stu.neu.edu.cn; 2 Kang Chiao International School, East China Campus, Kunshan 215332, China; kcismark@gmail.com

**Keywords:** graphene, iridium oxide, water electrolysis, oxygen evolution reaction, electrocatalyst

## Abstract

Producing hydrogen by water electrolysis has attracted significant attention as a potential renewable energy solution. In this work, a catalyst with reduced graphene oxide (rGO) loaded on IrO_2_/TiO_2_ (called rGO/IrO_2_/TiO_2_) was designed for the catalytic oxygen evolution reaction (OER). The catalyst was synthesized by coating graphene oxide onto a pretreated IrO_2_/TiO_2_ precursor, followed by thermal treatment at 450 °C to achieve reduction and the adhesion of graphene to the substrate. The graphene support retained its intact sp^2^ carbon framework with minor oxygen-containing functional groups, which enhanced electrical conductivity and hydrophilicity. Benefiting from the synergistic effect of an rGO, IrO_2_, and TiO_2_ matrix, the rGO/IrO_2_/TiO_2_ catalyst only needed overpotentials of 240 mV and 320 mV to reach 10 mA cm^−2^ and 100 mA cm^−2^ in the OER, along with excellent stability over 50 h. Its morphology and crystalline structure were characterized by SEM and XRD spectroscopy, and its electrochemical performance was tested by LSV analysis, EIS impedance spectrum, and double-layer capacitance (C_dl_) measurements. This work introduces an innovative and eco-friendly strategy for constructing a high-performance, functionalized Ir-based catalyst.

## 1. Introduction

Currently, fossil fuels are the primary sources of hydrogen production due to their efficiency and cost-effectiveness [1]. However, with the rising demand for hydrogen as a purely clean energy source and growing concerns about the environmental impact of fossil fuels, such as global warming, it has become imperative to develop sustainable and economical methods for large-scale hydrogen production [2,3]. Currently, hydrogen produced by electrocatalytic water splitting using sustainable electricity is considered an ideal alternative energy source because it is pollution-free and carbon-free in both its production and utilization stages [4,5].

As shown in Equation (1), water electrolysis, or water splitting, is an endothermic reaction and has a ΔG of 237.1 kJ mol^−1^ under standard conditions (25 °C, 101.325 kPa), indicating a non-spontaneous process. Water electrolysis involves two half-reactions: the hydrogen evolution reaction (HER) at the cathode and the oxygen evolution reaction (OER) at the anode. Among the different electrolysis conditions, alkaline conditions are particularly advantageous for an OER due to their ability to enhance catalyst stability and performance [6]. Alkaline media also offer higher tolerance to corrosion and degradation, making them suitable for long-term industrial applications [7]. In these conditions, water molecules at the cathode gain electrons to produce hydrogen, and the remaining hydroxide anions transport to the anode to lose electrons and produce oxygen, as shown in Equations (2) and (3).

Water splitting: 2H_2_O (l)→2H_2_ (g) + O_2_ (g) (1)

In alkaline electrolyte:

HER: 2H_2_O (l) + 4e^−^→2H_2_ (g) + 4OH (2)

OER: 4OH^−^→O_2_ (g) + 2H_2_O (l) + 4e^−^ (3)

The HER entails a two-electron transfer, while the OER necessitates a four-electron transfer, which requires the reaction to surmount a higher energy barrier. Consequently, individuals are dedicated to discovering exceptional OER electrocatalysts to lower the energy barrier of water electrolysis [8]. Under standard conditions, the theoretical potential to run the water electrolysis reaction is 1.23 V (0 V for HER and 1.23 V for OER, vs. RHE) [9]. However, the actual voltage required for water electrolysis is much higher than the theoretical value due to several factors, including energy barriers related to electrochemical kinetics, mass transport limitations, and resistance arising from the electrode–electrolyte interface [10]. These extra voltage demands are also known as overpotential. Therefore, the actually applied potential (voltage) for water electrolysis (*E*_ap_) can be determined using Equation (4), where η_HER_ and η_OER_ are overpotentials and iR is the voltage drop due to system resistances.

*E*_ap_ = 1.23 V + η_HER_ + η_OER_ + iR (4)

To reduce the overpotential of an OER, many chemists have attempted to develop high-efficiency OER catalysts, such as noble metals Pt [11], Ru [12], Ir [13], and Pd [14], and their oxides, as well as some transition metal-based alloys such as Ni-Fe [15], Ni-Cu [16], Ni-Co [17], and Ni-Ir [18]. Currently, Ir-based catalysts are considered promising due to their excellent OER performance and stability [19]. However, there is still room to further enhance their electrocatalytic performance. For improvement, Zhang et al. synthesized a Ru@Ir-O catalyst by introducing a core–shell nanostructure with oxygen bound to the Ir shell, leading to an increase in activity and resulting in an extremely low OER overpotential of 238 mV (at 10 mA cm^−2^) [20]. Zhu et al. prepared the Ir-Co_3_O_4_ catalyst by doping dispersed Ir atoms into spinel Ir-Co_3_O_4_. The introduction of Ir single atoms increased electronic conductivity and decreased the adsorption energy barrier, resulting in an overpotential of 236 mV (at 10 mA cm^−2^) [21].

In addition to enhancing the intrinsic activity of electrocatalysts, increasing the number of active sites—regions on the surface of a catalyst that adsorb and desorb reactants to catalyze the reaction—can also improve OER performance, as demonstrated by measuring the electrochemical surface area (ECSA) [22]. Reduced graphene oxide (rGO), a material with an exceptional surface area and electrical conductivity, can provide more catalytically active sites [23]. Moreover, despite the nitrogen gas reduction process that eliminates oxygen-containing functional groups from graphene oxide (GO) and creates large defect regions that enhance electron transfer, carbonyl groups (C=O) at the edges of GO nanosheets may still remain and serve as active sites by adsorbing intermediate species for water splitting [24]. These properties make rGO a promising material for further enhancing OER catalysts. Huang et al. developed an rGO-coated Ni_3_Se_2_ catalyst on nickel foam, denoted as rGO/Ni_3_Se_2_/NF, which provided abundant active sites due to rGO integration and demonstrated excellent OER and HER performances with low overpotentials of 292.61 mV and 251.01 mV, respectively, at ±10 mA cm^−2^ [25]. Bhosale et al. developed a vanadium oxide-reduced graphene oxide–nickel oxide (VrG/NiO) electrocatalyst that utilizes active sites at the interface of V_2_O_5_, rGO, and NiO to achieve an efficient oxygen evolution reaction (OER) performance with a low overpotential of 155.47 mV at 10 mA cm^−2^ [26].

Even though they have achieved good performance, the potential benefits of integrating rGO and IrO_2_ have yet to be fully realized.

Herein, a reduced graphene oxide (rGO)-coated IrO_2_ catalyst on TiO_2_ (denoted as rGO/IrO_2_/TiO_2_) was designed by solution coating and then calcination at 450 °C for a catalytic oxygen evolution reaction (OER). Through the synergetic effect of rGO and IrO_2_, the overpotential decreased to 240 mV and 320 mV at 10 mA cm^−2^ and 100 mA cm^−2^ in the OER, respectively. Accordingly, it exhibits a low Tafel slope and large double-layer capacitance (C_dl_) values. This article highlights the impact of rGO on IrO_2_ for OER performance, providing innovative insight for developing highly efficient Ir-based catalysts.

## 2. Experimental Methods

### 2.1. Chemicals

Every material was commercially obtainable: N-propanol (CH_3_CH_2_CH_2_OH, 99.5%) and oxalic acid (H_2_C_2_O_4_, 99.5%) were procured from Macklin (Shanghai, China), and KOH (99.5%) was purchased from Sinopharm (Beijing, China). H_2_IrCl_6_·6H_2_O (99.5%) was procured from Johnson Matthey (London, UK). Chemicals were utilized as provided, without an additional purification process.

### 2.2. Characterization Methods

The structural analysis and crystalline phase purity of all samples were examined by X-ray diffraction (XRD) on a Panalytical Empyrean diffractometer (Almelo, The Netherland) utilizing Cu Kα radiation spectra (λ = 1.54 Å, 300 W) in the 2θ range of 20–80°. Scanning electron microscopy (SEM) and energy dispersive spectroscopy (EDS) mapping were performed before and after 50 h, testing for structural morphologies and elemental analysis at 5 kV using a Tescan MAIA3 XMH (15 kV) (Kohoutovice, Czech Republic). Furthermore, Raman spectroscopy was conducted using a Renishaw (Gloucestershire, UK) inVia Raman microscope to analyze vibrational modes and confirm the presence of carbon-based structures. X-ray photoelectron spectroscopy (XPS) was performed on a Shimadzu AXIS SUPRA+ instrument (Kyoto, Japan). The charge was corrected by the C1s = 284.8 eV binding energy standard, and elemental binding energy was analyzed based on the confirmation of C1s at 284.8 eV.

### 2.3. Electrochemical Measurements

The electrochemical performance of the proposed catalysts was evaluated using the electrochemical workstation CH Instrument Ins (CHI 660) (Austin, TX, USA). A conventional three-electrode setup was employed, consisting of a Pt foil as the counter electrode, an Hg/HgO reference electrode (0.098 V vs. SHE), and rGO/IrO_2_/TiO_2_ and IrO_2_/TiO_2_ as the working electrodes for all electrochemical measurements. Catalytic activity was assessed in a 1 M KOH electrolyte (pH = 14) at room temperature (25 °C). The working potential of samples was converted from E (V vs. SHE) to E (V vs. RHE) using the Nernst equation—Equation (5):

(5) $$E_{\left(V\;vs.\;RHE\right)}=E_{Hg/HgO}^{\theta }+E_{Hg/HgO}+0.059\times pH-iR$$

where $E_{Hg/HgO}^{\theta }$ is 0.098 V, $E_{Hg/HgO}$ is the measured potential, and the pH is 14. In addition, the current (*i*/A) was directly converted into current density (*j*/mA cm^−2^), as the electrodes had a reacting surface area of 1 cm^2^. All polarization curves were corrected by iR compensation, where the potential values were corrected for the IR drop by subtracting the voltage loss due to the ohmic resistance of the electrolyte.

### 2.4. Synthesis of IrO_2_/TiO_2_

**Figure 1:**
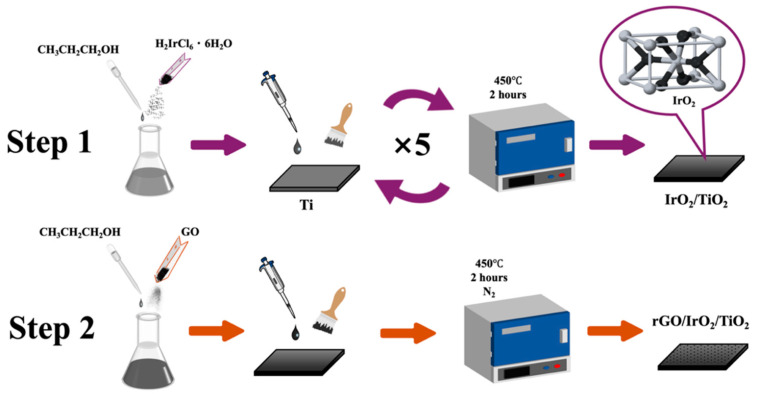
Schematic of rGO/IrO_2_/TiO_2_ synthesis procedure.

First, a solution of 1-propanol and chloroiridinic acid with a mass ratio of 14:1 was prepared and stirred thoroughly by a magnetic stirrer (15 min). Using a pipette and an analytical balance, 0.3 g of this solution was added to a 10 × 10 × 0.1 cm^3^ pure Ti plate that had been acid-etched with 10% HCl (30 min), and was spread evenly on the plate using a brush that had been dipped in the same solution. After the solution dried, the plate was calcined in the atmosphere furnace at 450 °C for 2 h. This process was repeated another 4 times on the same Ti plate, as this could allow the IrO_2_ to be more evenly distributed and result in fewer cracks between IrO_2_ particles on the plate. Additionally, the surface of the Ti plate produced titanium dioxide during calcination. As a result, an IrO_2_ catalyst with approximately 1.0 mg/cm^2^ of Ir was synthesized. A detailed schematic illustration of the synthesis procedure is depicted as Step 1 in Figure 1.

### 2.5. Synthesis of rGO/IrO_2_/TiO_2_

Another solution of 1-propanol and graphene oxide powder with a mass ratio of 11:1 was prepared and stirred thoroughly by a magnetic stirrer (15 min). Next, 0.3 g of this solution was added to the IrO_2_/TiO_2_ and was applied evenly using a brush that had been dipped in the same solution. After the solution dried, the plate was calcined in nitrogen at 450 °C for 2 h, ensuring that the GO reduced successfully. After that, the synthesis of rGO/IrO_2_/TiO_2_ with approximately 0.2 mg/cm^2^ of graphene was finished (Step 2 in Figure 1), and both the IrO_2_/Ti and the rGO/IrO_2_/TiO_2_ were sliced into 1 × 1.5 × 0.1 cm^3^ samples to prepare for the experiment (Figure S1).

## 3. Results and Discussion

### 3.1. Material Characterizations

**Figure 2:**
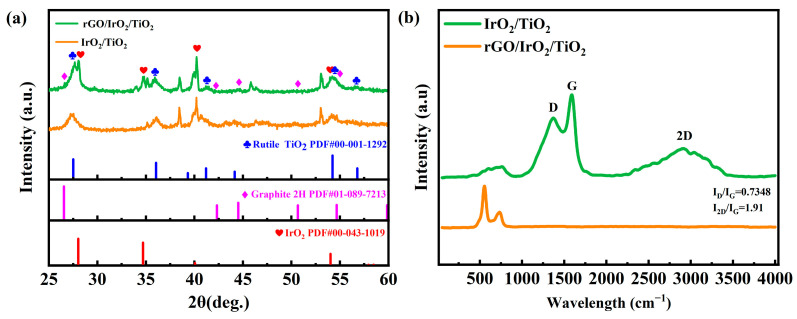
Crystal and structural characterization of catalysts IrO_2_/TiO_2_ and rGO/IrO_2_/TiO_2_. (**a**) XRD analysis and (**b**) Raman spectra.

As shown in Figure 2a, the phase structure and crystallinity of catalysts IrO_2_/TiO_2_ and rGO/IrO_2_/TiO_2_ were investigated through XRD. XRD spectra of the catalyst IrO_2_/TiO_2_ demonstrated characteristic peaks at 28.04°, 34.79°, 40.19°, and 53.99°, matching the (110), (101), (200), and (211) planes of the IrO_2_ (PDF#00-043-1019), respectively. Likewise, XRD spectra of the catalyst rGO/IrO_2_/TiO_2_ demonstrated characteristic peaks at 27.64°, 35.06°, 40.14°, and 54.11°, also matching the (110), (101), (200), and (211) planes of the IrO_2_ (PDF#00-043-1019), respectively. This indicates that both catalysts contained IrO_2_. XRD spectra of the catalyst IrO_2_/TiO_2_ also demonstrated characteristic peaks at 27.62, 35.98, 41.20, 54.28, and 56.63, matching the (110), (101), (111), (211), and (220) of the rutile TiO_2_ (PDF#00-001-1292), respectively. Similarly, XRD spectra of the catalyst rGO/IrO_2_/TiO_2_ demonstrated characteristic peaks at 27.31, 36.09, 41.24, 54.37, and 56.65, matching the (110), (101), (111), (211), and (220) of the rutile TiO_2_ (PDF#00-001-1292), respectively. This suggests that the surfaces of the Ti plates oxidized to TiO_2_. However, only the XRD spectra of the catalyst rGO/IrO_2_/TiO_2_ demonstrated little characteristic peaks at 26.66, 42.15, 44.60, 50.65, and 54.73, corresponding to the (002), (100), (101), (102), and (004) of the graphite (PDF#00-043-1019), respectively. This justified the coated rGO in the catalyst.

According to Figure 2b, Raman spectra of bare IrO_2_/TiO_2_ and rGO/IrO_2_/TiO_2_ samples provide clear evidence of successful graphene deposition and offer insight into the structural quality of the rGO layer. The IrO_2_/TiO_2_ control exhibits a featureless baseline in the 1000–3000 cm⁻^1^ region, confirming the absence of graphitic carbon. Raman peaks at 554.49 cm^−1^ and 733.47 cm^−1^ are attributed to the characteristic vibrational modes of iridium oxide (IrO_2_). In contrast, the rGO/IrO_2_/TiO_2_ composite displays the three hallmark bands of graphene-based materials: the D band at 1371.50 cm^−1^, the G band at ~1590.09 cm^−1^, and the 2D band near 2916.70 cm^−1^. The D band, arising from the breathing modes of sp^2^ carbon rings activated by defects and edge sites, and the G band, corresponding to the in-plane E_2_g vibration of sp^2^ carbon networks, are both broadened relative to those of pristine graphene, reflecting residual oxygen functionalities and interfacial interactions with the IrO_2_/TiO_2_ substrate [27]. The intensity ratio I_D_/I_G_ of approximately 0.73 indicates a moderate defect density—typical for reduced graphene oxide—that is advantageous for catalysis because defect sites can serve as additional active centers for electron transfer. Meanwhile, the high I_2D_/I_G_ ratio of 1.91 is characteristic of few-layer graphene, rather than bulk graphite, and confirms that thermal reduction preserved a relatively thin graphene coating [28]. A slight upshift in the G peak further suggests a charge transfer between the rGO and the underlying oxide, consistent with the enhanced electrical conductivity and accelerated OER kinetics observed in electrochemical measurements. Together, these Raman features validate the presence of a defect-rich, few-layer rGO film whose structural and electronic characteristics underpin the superior electrocatalytic performance of the rGO/IrO_2_/TiO_2_ composite.

**Figure 3:**
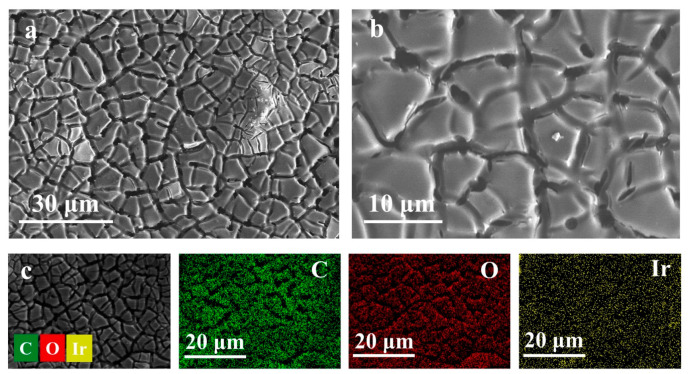
Morphology and structure characterizations. (**a**,**b**) SEM images of rGO/IrO_2_/TiO_2_ at different magnifications and (**c**) EDS mappings of rGO/IrO_2_/TiO_2_.

The surface morphologies of IrO_2_/TiO_2_ and rGO/IrO_2_/TiO_2_ were examined using a scanning electron microscope (SEM) (Figure 3 and Figure S2). Figure 3a,b present SEM images of rGO/IrO_2_/TiO_2_ at different magnifications for comparison. The surface morphology of rGO/IrO_2_/TiO_2_ displays a cracked mud-like structure comparable to that of IrO_2_/TiO_2_, yet with discernible distinctions. The edges of the polygonal platelets exhibit a slight degree of smoothness and definition, indicative of a thin graphene layer coating the IrO_2_ structure. At higher magnification (Figure 3b), the surface of the platelets displays a more textured appearance relative to that of IrO_2_/TiO_2_, which can be attributed to the graphene coating.

As illustrated in Figure 3c, EDS mappings of rGO/IrO_2_/TiO_2_ provide insight into its elemental composition following the graphene coating process. In addition to Ir and O, there was a notable increase in the carbon signal, which was discernible and distributed uniformly across the surface. This result corroborates the successful deposition of the graphene layer onto IrO_2_/TiO_2_. Consequently, the distribution of Ir, O, and C appeared to be uniform and followed the pattern of the cracked electrode surface.

Scanning electron microscopy (SEM) imaging revealed a distinct cracked mud-like structure for both catalysts, with rGO/IrO_2_/TiO_2_ exhibiting a smoother surface texture, indicative of the graphene coating. Energy-dispersive X-ray spectroscopy (EDS) mapping further confirmed the successful deposition of the graphene layer on the IrO_2_/TiO_2_ surface. While the overall cracked mud-like structure was maintained, the graphene coating introduced subtle changes to the surface texture and significantly increased the carbon content of the electrode. These structural modifications likely played a crucial role in the enhanced electrocatalytic properties observed for rGO/IrO_2_/TiO_2_, as discussed in subsequent sections.

**Figure 4:**
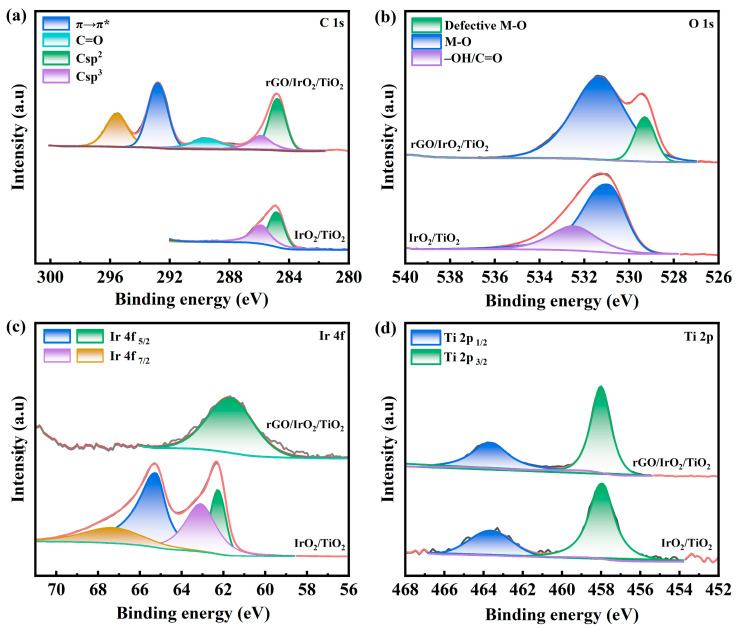
High-resolution XPS spectra of rGO/IrO_2_/TiO_2_ and IrO_2_/TiO_2_ for (**a**) C 1s, (**b**) O 1s, (**c**) Ir 4f, and (**d**) Ti 2p.

Further X-ray photoelectron spectroscopy (XPS) analysis was conducted to determine the elemental composition and chemical states of the samples. The XPS survey spectrum (Figure 4 and Figure S3) confirmed the presence of C, O, Ir, and Ti, indicating the successful synthesis of rGO/ IrO_2_/TiO_2_. The C 1s XPS spectrum (Figure 4a) exhibited five characteristic peaks at 295.53, 292.81, 289.52, 284.08, and 285.89 eV, corresponding to the Auger peak of Ir, the conjugated structure (π→π*) of graphene, sp^2^-hybridized graphitic carbon, and sp^3^-hybridized carbon, respectively. Compared with that of IrO_2_/TiO_2_, the C 1s spectrum of rGO/IrO_2_/TiO_2_ displayed distinct graphene-related peaks, along with a weakened C=O peak, confirming the presence of reduced graphene oxide (rGO) while maintaining the integrity of the graphene framework. The deconvoluted O 1s XPS spectrum (Figure 4b) revealed two peaks of rGO/IrO_2_/TiO_2_ at 531.34 and 529.3 eV, and two peaks of IrO_2_/TiO_2_ at 532.49 and 530.96 eV. The incorporation of reduced graphene oxide (rGO) induced metal–oxygen bond reconstruction, while interfacial electron transfer promoted oxygen vacancy formation. As shown in Figure 4c, the Ir 4f XPS spectrum exhibited two characteristic peaks corresponding to 4f₇/_2_ and 4f_5/2_ [29]. Notably, rGO/ IrO_2_/TiO_2_ displayed only a single oxidation state (4f_5/2_), unlike IrO_2_/TiO_2_. The Ti 2p XPS spectrum (Figure 4d) showed two peaks assigned to 2p_1/2_ and 2p_3/2_, confirming the structural integrity and stability of the TiO_2_ substrate before and after rGO loading. These findings demonstrate that loading rGO onto IrO_2_/TiO_2_ not only introduced graphene-like catalytic properties, but also served as a structural support layer, significantly improving the electrode’s electronic conductivity. Moreover, IrO_2_ and TiO_2_ remained uniformly distributed without forming low-valence states or alloys.

### 3.2. Electrocatalytic Properties

**Figure 5:**
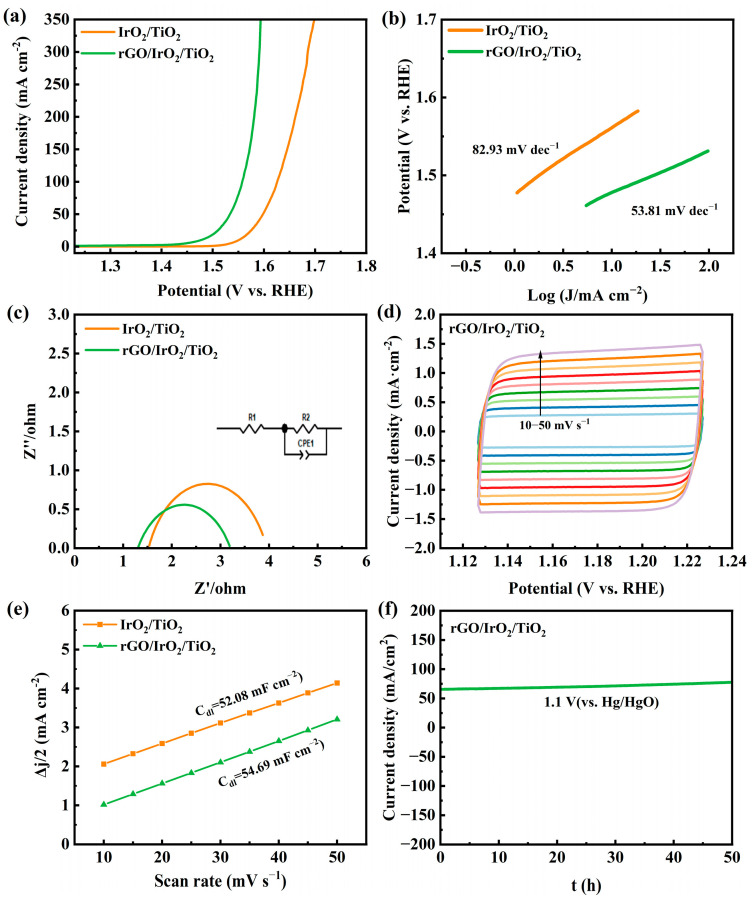
(**a**) Polarization curves; (**b**) corresponding Tafel plots in 1.0 M KOH electrolyte of IrO_2_/TiO_2_ and rGO/IrO_2_/TiO_2_; (**c**) EIS Nyquist plots; (**d**) CV curves at 10 to 50 mV s^−1^ scan rates; (**e**) double-layer capacitance (Cdl) values; and (**f**) stability test for rGO/IrO_2_/TiO_2_.

A conventional three-electrode electrochemical system was employed to assess the electrocatalytic oxygen evolution reaction (OER) activity of the resulting catalyst in an alkaline solution to ascertain the electrocatalytic performance. Figure 5a shows the polarization curves of each sample. It can be observed that the OER catalytic activity was significantly enhanced after the graphene layer was coated, where its overpotentials at 10 and 100 mA cm^−2^ were only 240 mV and 320 mV, respectively, compared to 320 mV and 400 mV for the original IrO_2_ catalyst at the same current density.

Figure 5e shows the Tafel slope of each tested electrode. It demonstrates that the rGO/IrO_2_/TiO_2_ had the smallest value of 53.81 mV dec^−1^, which was less than IrO_2_/TiO_2_’s 82.93 mV dec^−1^. The lower Tafel slope indicated a more rapid reaction kinetics and a more favorable OER mechanism, which was likely associated with the efficient adsorption and desorption of oxygen intermediates, facilitating the four-electron transfer pathway commonly observed for iridium-based catalysts.

Electrochemical impedance spectroscopy (EIS) measurements coupled with equivalent circuit modeling were employed to more accurately elucidate the kinetics of the oxygen evolution reaction (OER) process. Figure 5c shows that the diameter of the semicircle for the rGO/IrO_2_/TiO_2_ electrode was notably smaller than that of pristine IrO_2_/TiO_2_, indicating that the rGO coating lowered the overall impedance and accelerated interfacial charge transfer. This reduced impedance corroborates the enhanced electron transport pathways provided by the conductive graphene network on the catalyst surface.

Figure 5e shows the capacitive current densities as a function of the scanning rate for each catalyst, which was extracted from the CV curves tested (Figure 5d and Figure S4). These results illustrate that the C_dl_ of the rGO/IrO_2_/TiO_2_ was 54.69 mF·cm^−2^, higher than the IrO_2_/TiO_2_’s 52.08 mF·cm^−2^, proving that graphene coating on electrode materials can increase their electrochemically active sites, which is a core measurement of the electrocatalytic property.

In addition, to evaluate the stability of the rGO/IrO_2_/TiO_2_ catalyst, a chronoamperometry test under the condition of 1.1 V (vs. Hg/HgO) was performed. As shown in Figure 5f, the change was very subtle within 50 h of continuous operation, indicating the excellent electrochemical stability of the catalyst.

**Figure 6:**
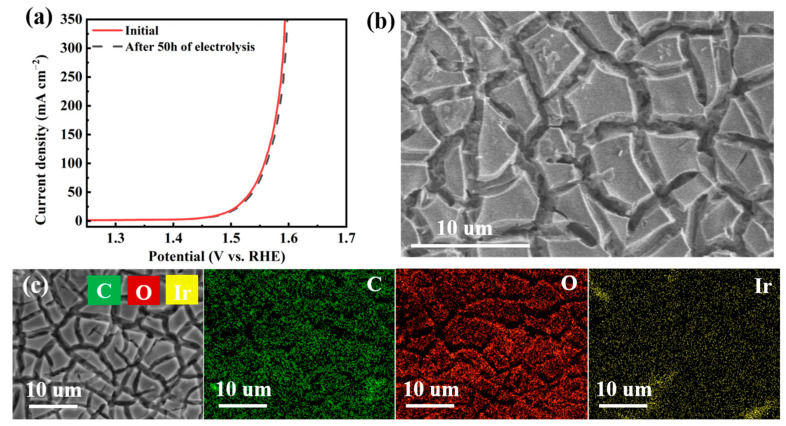
(**a**) Polarization curves of the rGO/IrO_2_/TiO_2_ catalyst after 50 h of continuous electrolysis in 1.0 M KOH; (**b**) SEM image of the electrode surface after the 50 h stability test (scale bar 10 µm); (**c**) corresponding EDS elemental maps of C (green), O (red) and Ir (yellow) after 50 h of electrolysis (scale bar 10 µm).

Figure 6a compares the LSV polarization curves of the rGO/IrO_2_/TiO_2_ electrode recorded initially and after 50 h of continuous OER testing in 1.0 M KOH. The overpotential required to reach 10 mA cm^−2^ increased only from 240 mV to 251 mV, and that for 100 mA cm^−2^ rose from 320 mV to 332 mV—corresponding to modest shifts of ~11 mV and ~12 mV, respectively. Such small potential increases demonstrate that the catalyst retained most of its activity under prolonged operation. SEM imaging after 50 h (Figure 6b) shows that the characteristic cracked “mud-like” morphology of the catalyst surface remained intact, and EDS maps (Figure 6c) confirm a uniform distribution of C, O, and Ir. Together, these results indicate that the rGO coating effectively preserved the structural integrity and catalytic performance of the IrO_2_/TiO_2_ electrode during extended alkaline electrolysis. For comparative evaluation of electrochemical performance, overpotentials at 10 and 100 mA cm^−2^ were benchmarked against similar catalysts in Table 1.

Electrochemical measurements showed that the rGO/IrO_2_/TiO_2_ catalyst exhibited superior performance. The enhanced performance of the rGO/IrO_2_/TiO_2_ catalyst can be ascribed to several contributing factors. The incorporation of rGO is believed to have augmented the overall electrical conductivity of the catalyst, thereby facilitating accelerated electron transfer during the OER process. The interaction between rGO and IrO_2_ may have created beneficial synergistic effects, potentially altering the electronic structure of the active sites and optimizing the adsorption energies of reaction intermediates. The elevated surface area of rGO furnished supplementary active sites for the OER, as substantiated by the augmented C_dl_ value. Moreover, the graphene coating may have safeguarded the underlying IrO_2_ from degradation, thereby enhancing the catalyst’s long-term stability.

## 4. Conclusions

In summary, this study demonstrated a promising strategy for enhancing the performance of Ir-based OER catalysts through the integration of reduced graphene oxide. The surface morphology of rGO/IrO_2_/TiO_2_ exhibited a comparable cracked mud-like structure, with a uniform dispersion of graphene across the surface. XPS analysis revealed that Ir existed in the +4 oxidation state (IrO_2_), while Ti was present as +4 (TiO_2_). The graphene support retained its intact sp^2^ carbon framework (C 1s at 284.8 eV) with minor oxygen-containing functional groups (C=O at 289.52 eV), which enhanced electrical conductivity and hydrophilicity. Raman spectroscopy (I_2D_/I_G_ = 1.91) further verified the defective structure of graphene, which facilitated metal oxide anchoring and charge transfer. The rGO/IrO_2_/TiO_2_ catalyst demonstrated exceptional catalytic activity. It only needed an overpotential of 240 mV to reach 10 mA cm^−2^ and 320 mV to reach 100 mA cm^−2^ in the OER, and had the smallest Tafel slope of 53.81 mV dec^−1^. Its double-layer capacitance (C_dl_) was 54.69 mF·cm^−2^, which further illustrates its large electrochemically active surface area. These findings contribute to the ongoing efforts to develop high-performance catalysts for water electrolysis and provide valuable insights for future research in this field.

## Figures and Tables

**Table 1:** Electrochemical properties.

Catalyst	Electrolyte	Overpotential at 10 mA cm^−2^ (mV)	Overpotential at 100 mA cm^−2^ (mV)	Reference
rGO/IrO_2_/TiO_2_	1 M KOH	240	320	This work
IrOx/TiO_2_ (10:90) composite	1 M KOH	300	—	Josep Boter-Carbonell et al. [30]
Core–shell IrO_2_@Ir	1 M KOH	255	—	Wenwu Zhong et al. [31]
Porous IrO_2_ (1:100) at 450 °C	0.5 M H_2_SO_4_	276	—	Guoqiang Li et al. [32]
IrO_2_/Ti foil annealed at 400 °C for 60 h	0.5 M H_2_SO_4_	282	391	Deng, Qian et al. [33]

## Data Availability

The original contributions presented in this study are included in the article/Supplementary Material. Further inquiries can be directed to the corresponding author.

## Supplementary Materials

The following supporting information can be downloaded at: https://www.mdpi.com/article/10.3390/molecules30092069/s1, Figure S1. Photographs of (a) rGO/IrO_2_/TiO_2_; (b) IrO_2_/TiO_2_. Figure S2. Morphology and structure characterizations. (a,b) SEM images of IrO_2_/TiO_2_ at different magnifications; (c) EDS mappings of IrO_2_/TiO_2_. Figure S3. Summary XPS spectra of rGO/IrO_2_/TiO_2_ and IrO_2_/TiO_2_. Figure S4. CV curves at 10 to 50 mV s^−1^ scan rates of IrO_2_/TiO_2_. Tabel S1. XPS test data related to rGO/IrO_2_/TiO_2_. Tabel S2. XPS test data related to IrO_2_/TiO_2_.
