# Supplementary material for: Reduced Graphene Oxide-Coated Iridium Oxide as a Catalyst for the Oxygen Evolution Reaction in Alkaline Water Electrolysis
Source: Molecules. 2025 May 7;30(9):2069. doi: 10.3390/molecules30092069 (PMC12073217; doi:10.3390/molecules30092069)
Supplement: Supplementary file 1 [file molecules-30-02069-s001.zip › molecules-3547074-supplementary.pdf]

Figure S1. Photographs of (a) $rGO/IrO_2/TiO_2$; (b) IrO_2/TiO_2.

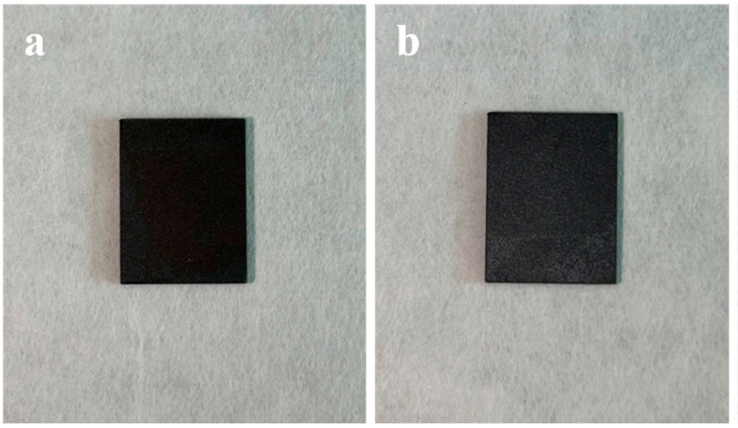

Figure S2. Morphology and structure characterizations. (a, b) SEM images of IrO_2/TiO_2 at different magnifications; (c) EDS mappings of IrO_2/TiO_2.

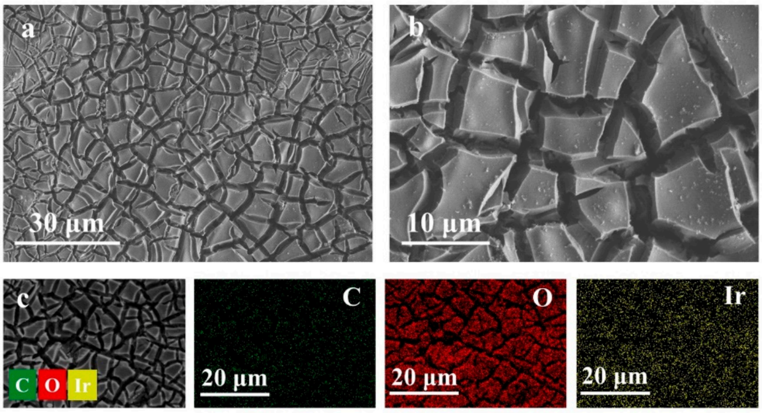

Figure S3. Summary XPS spectra of $rGO/IrO_2/TiO_2$ And IrO_2/TiO_2.

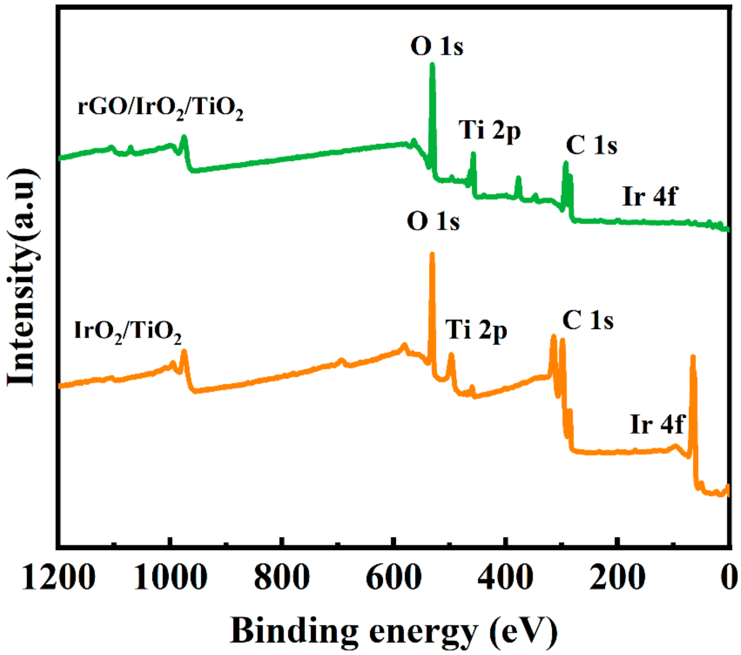

Figure S4. CV curves at 10 to 50 mV s^{-1} scan rates of IrO_2/TiO_2.

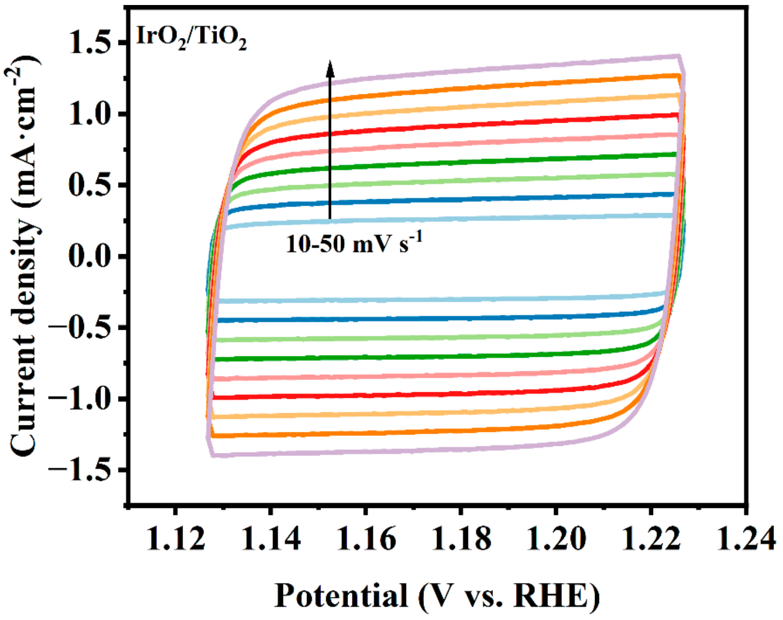

Tabel S1. XPS test data related to $rGO/IrO_2/TiO_2$.

		Fitted peak position	FWHM eV	Area	Atomic %
C 1s	-	295.53	1.7	63793.96	0.62
	π-π*	292.81	1.55	108366.97	6.76
	C=O	289.52	2.4	32574.89	11.47
	C sp^3	285.89	1.33	73968.87	3.44
	C sp^2	284.8	1.67	30735.86	7.79
Ti 2p	Ti 2p 1/2	463.61	2.16	51762.93	6.16
	Ti 2p 3/2	458.01	1.18	89199.6	10.58
O 1s	M-O	531.34	2.8	329160.74	41.28
	Defective M-O	529.3	1.08	69143.94	8.66
Ir 4f	Ir 4f 5/2	61.67	2.59	6684.54	0.62

Tabel S2. XPS test data related to IrO_2/TiO_2.

		Fitted peak position	FWHM eV	Area	Atomic %
C 1s	C sp^2	284.8	1.23	45876.18	4.7
	C sp^3	285.85	1.5	39777.05	4.08
Ti 2p	Ti 2p 3/2	465.63	2.56	11622.17	1.35
	Ti 2p 1/2	459.93	1.5	24529.42	2.83
O 1s	-OH/C=O	532.49	2.45	93531.57	11.42c
	M-O	530.96	2.21	243898.42	29.74
Ir 4f	Ir 4f 5/2	65.23	1.57	215855.83	19.41
	Ir 4f 7/2	62.27	0.84	79789.07	7.16
	Ir 4f 5/2	63.1	1.73	134695.1	12.1
	Ir 4f 7/2	67.28	3.36	80223.39	7.22
